# Topographic and surgical risk factors for high postoperative residual astigmatism after small incision lenticule extraction in patients with different degrees of myopia: a retrospective cohort study

**DOI:** 10.1186/s12886-024-03296-x

**Published:** 2024-01-29

**Authors:** Chia-Yi Lee, Jen-Hsiang Shen, Chen-Cheng Chao, Ie-Bin Lian, Jing-Yang Huang, Shun-Fa Yang, Chao-Kai Chang

**Affiliations:** 1 Institute of Medicine, Chung Shan Medical University, Taichung, Taiwan; 2 Nobel Eye Institute, No. 13-5, Gongyuan Rd., Zhongzheng Dist, 100008 Taipei, Taiwan; 3 Department of Ophthalmology, Jen-Ai Hospital Dali Branch, Taichung, Taiwan; 4 Department of Optometry, Nursing, and Management, MacKay Junior College of Medicine, Taipei, Taiwan; 5 Institute of Statistical and Information Science, National Changhua University of Education, Changhua, Taiwan; 6 Department of Medical Research, Chung Shan Medical University Hospital, Taichung, Taiwan; 7 Department of Optometry, Da-Yeh University, Changhua, Taiwan

**Keywords:** Small incision lenticule extraction, Astigmatism, Corneal curvature, Topography, Myopia

## Abstract

**Background:**

To evaluate the possible topographic and surgical risk factors for high postoperative residual astigmatism in patients who undergo small-incision lenticule extraction (SMILE) surgery and have different myopia degrees.

**Methods:**

A retrospective cohort study was conducted, and patients who underwent SMILE surgery were enrolled. A total of 80 and 150 eyes from 40 to 75 individuals, respectively, were selected as the low myopia and high myopia groups. The demographic data, visual acuity, refraction, topographic parameters and surgical settings were recorded. Multiple linear regression with interaction tests were performed to survey the risk factors for high postoperative residual astigmatism in each group.

**Results:**

Five (6.25%) and 9 (6.00%) eyes presented with high postoperative residual astigmatism in the low myopia and high myopia groups, respectively, but these differences were not significant (*P* = 0.569). A steep corneal curvature was correlated with a greater risk of high postoperative residual astigmatism in the low myopia group (*P* = 0.015), while a higher degree of cycloplegic cylinder power, steeper corneal curvature, greater topographic cylinder power, smaller optic zone and longer incision length were associated with a high rate of postoperative residual astigmatism in the high myopia group (all *P* < 0.05). In addition, the interaction effects of cycloplegic and topographic cylinder power and longer incision length on the incidence of high postoperative residual astigmatism development were more evident in the high myopia group than in the low myopia group (all *P* < 0.05).

**Conclusions:**

A steep corneal curvature correlates with a high risk of high postoperative residual astigmatism after SMILE surgery, and a higher degree of cycloplegic and topographic cylinder and longer incision are associated with high postoperative residual astigmatism in individuals with high myopia.

## Background

Refractive surgeries have been performed to correct myopia and astigmatism for more than 20 years [1]. Photorefractive keratectomy, laser in situ keratomileusis (LASIK), and small incision lenticule extraction (SMILE) are refractive surgeries that are commonly performed worldwide [2]. The number of individuals scheduled for SMILE surgery has recently increased, which may be due to the decreased risk of dryness or other ocular symptoms [3]. Concerning the LASIK and SMILE procedures, the efficiency and predictability of both surgery methods are analogous [4, 5], and SMILE surgery results in better postoperative corneal sensitivity than LASIK surgery [6].

Despite the acceptable safety of refractive surgeries, postoperative complications can develop after refractive surgery [7]. The postoperative complications of refractive surgery include dry eye disease, epithelial ingrowth, diffuse lamellar keratitis, superficial keratitis, infectious keratitis and corneal ectasia [8, 9], and myopic regression is a natural course and a common postoperative complication of refractive surgery [10–12]. In addition, postoperative astigmatism after refractive surgery can occur, and LASIK is associated with favorable outcomes [13, 14]. Postoperative astigmatism after refractive surgery can lead to decreased vision, and LASIK surgery is not uncommonly performed as a secondary enhancement procedure for correcting postoperative astigmatism [15].

The possible risk factors for post-LASIK astigmatism include small optic zone and high preoperative astigmatism [16]. Additionally, high preoperative astigmatism is correlated with high postoperative astigmatism in SMILE patients [17]. However, the topographic or surgical risk factors for high postoperative residual astigmatism after SMILE surgery have not been identified. Moreover, the risk factors for postoperative refractive error differ among patients who undergo LASIK surgery to correct different degrees of myopia [10, 16], and the risk factors for high postoperative residual astigmatism after SMILE surgery for different degrees of myopia may also differ and need further investigation.

As a consequence, the aim of the present study was to evaluate the risk factors for high postoperative residual astigmatism after SMILE surgery in patients with different degrees of myopia. The refractive, topographic and surgical factors were included in the analysis.

## Methods

### Participant selection

A retrospective cohort study was conducted at the Nobel Eye Clinic, Taipei Branch. The Nobel Eye Clinic was treated by a clinical group that mainly performed cataract and refractive surgeries in northern Taiwan. The participants who were enrolled in the present study met the following criteria: (1) aged 18 to 55 years, (2) underwent surgery at the Taipei Nobel Eye Clinic, (3) had spherical myopia for at least − 1.00 diopter (D), and (4) were followed up at any branch of the Nobel Eye Institute for at least one year. No inclusion criteria for the degree of astigmatism were set. Moreover, patients with uncorrected visual acuity (UCVA) worse than 20/4000 on the Snellen chart were excluded from the present study. Patients with a spherical equivalent refractive error less than − 6.00 D according to cycloplegic refraction were enrolled in the high myopia group, and the remaining participants were enrolled in the low myopia group. A total of 80 and 150 eyes from 40 to 75 patients were included in the low myopia and high myopia groups, respectively.

### Surgical technique

All the SMILE surgeries in the present study were performed by one experienced refractive specialist (C.-K.C.), and the nomogram for astigmatism was the same since all the SMILE surgeries were performed by C.-K.C. The SMILE surgery was performed with one femtosecond laser device (Visuamax 500, Carl Zeiss, Göschwitzer Str., Jena, Germany). The optic zone was created to be 5.5–6.9 mm according to the lenticule thickness and pupil size of each patient, and the corneal incision was created to be 3.0 mm at 105 degrees. After the angle kappa was confirmed via surgical microscopy via the coaxial sighted corneal light reflex method, the cornea was fixed by a suction ring, after which the surgeon triggered the femtosecond laser. After femtosecond laser radiation, the surgeon used a spatula and dissected the upper and lower interfaces of the lenticule, and the lenticule was removed with forceps. Postoperatively, levofloxacin eye drops, prednisolone suspensions and artificial tears were instilled for one week, followed by sulfamethoxazole and fluorometholone eye drops for approximately three weeks.

### Ophthalmic examination

All participants who underwent SMILE underwent similar ophthalmic examinations at the Taipei Nobel Eye Clinic. The preoperative exams included manifest refraction with best corrected visual acuity (BCVA) measurements, intraocular pressure measurements via pneumatic tonometry (NT-530, NIDEK Co., Ltd., Gamagori, Aichi, Japan), cycloplegic refraction measurements of sphere and cylinder powers via an autorefractor (KR-8900, Topcon, Itabashi-ku, Tokyo, Japan), central corneal thickness (CCT) with the corneal apex and thinnest part, corneal curvature, index of height decentration (IHD), index of surface variance (ISV), angle kappa, and corneal cylinder power examination via a topographic instrument (Oculus Pentacam, OCULUS Optikgeräte GmbH, Münchholzhäuser, Wetzlar, Germany). Postoperative data, which included UCVA, BCVA, and sphere and cylinder powers for measuring cycloplegic refraction, were collected one year after SMILE surgery. In addition, surgical indices, including the optic zone, cap thickness, and corneal lenticule thickness, were measured during SMILE surgery. The spherical equivalent (SE) in the present study was defined as the sphere power plus half of the cylinder power, and the difference in CCT was regarded as the CCT of the apex minus the CCT of the thinnest area in our study. A higher cycloplegic cylinder and high postoperative residual astigmatism were defined as a cycloplegic cylinder power equal to or greater than − 1.25 D in the present study, and a steep corneal curvature was defined as a mean corneal curvature greater than 45 D. Additionally, an incision length longer than 3.5 mm was set as a long incision length, and an optical zone larger than 6.5 mm was regarded as a large optical zone.

### Statistical analysis

SPSS version 20.0 (SPSS, Inc., Chicago, Illinois, USA) was used for the statistical analyses in our study. The Shapiro–Wilk test was used to check for a normal distribution in the low myopia and high myopia groups, and the results revealed that the two groups were normally distributed. Descriptive analysis was also performed to evaluate age, sex, preoperative refractive status, preoperative topographic parameters, and surgical parameters, and an independent t test was subsequently used to evaluate the differences in the above parameters between the two groups. Independent t tests were also adopted to evaluate the difference in surgical efficacy and predictability between the low myopia and high myopia groups at one year after surgery. A bar chart was drawn to show the efficiency and predictability of the various treatments between groups. The chi-square test was applied to analyze the distribution of high postoperative residual astigmatism between the two groups and the change in the cylinder axis after SMILE surgery. The multiple linear regression was adopted to calculate the correlation between postoperative residual astigmatism (as continuous variable) and age, sex, IOP, refractive indices including topographic parameters, such as steep or flat corneal curvature and cylinder axis (with-the-rule vs. against-the-rule), and surgical data in the whole study population, and yielded the coefficient with 95% confidence intervals (CI) for each variable. Then the multiple linear regression was used again to determine the coefficient with 95%CI for all the variables mentioned above supporting the development of high postoperative residual astigmatism in both the high and low myopia groups. The interaction test was subsequently run to evaluate the prominent risk factors for high postoperative residual astigmatism in both the high myopia group and the low myopia group according to the results of multiple linear regression. A *P* value < 0.05 was considered to indicate high statistical significance, and a *P* value lower than 0.001 was depicted as *P* < 0.001 in the present study.

## Results

The mean ages of the patients were 31.91 ± 6.76 years and 32.71 ± 6.43 years in the low myopia group and high myopia group, respectively, and these differences were not significant (*P* = 0.380). SE and sphere power, via either manifest or cycloplegic methods, were significantly greater in the high myopia group than in the low myopia group (all *P* < 0.001). In addition, the high myopia group also had a thicker CCT at the apex, thicker CCT at the thinnest region, greater lenticule thickness and a smaller optic zone than the low myopia group (all *P* < 0.05). The remaining preoperative features of the two groups are presented in Table 1.

**Table 1:** Preoperative features of the study population

Features (mean ± SD)	Low myopia group (*N* = 80)	High myopia group (*N* = 150)	***P***
Age	31.91 ± 6.76	32.71 ± 6.43	0.380
Sex (male: female)	17: 23	28: 47	0.692
Cycloplegic refraction			
SE	-4.07 ± 0.83	-7.35 ± 1.49	< 0.001*
Sphere	-3.58 ± 0.79	-6.78 ± 1.37	< 0.001*
Cylinder	-0.98 ± 0.73	-1.12 ± 0.86	0.222
IOP	18.03 ± 2.59	18.32 ± 2.52	0.405
BCVA	0.99 ± 0.04	0.99 ± 0.04	0.923
Manifest refraction			
SE	-4.12 ± 0.89	-7.18 ± 1.37	< 0.001*
Sphere	-3.64 ± 0.79	-6.67 ± 1.28	< 0.001*
Cylinder	-0.95 ± 0.74	-1.03 ± 0.79	0.477
Steep stimulated keratometry	44.02 ± 1.53	44.08 ± 1.60	0.785
Flat stimulated keratometry	42.51 ± 1.41	42.69 ± 1.47	0.377
Topographic cylinder	1.51 ± 0.76	1.40 ± 0.72	0.248
CCT			
Apex	557.25 ± 26.03	569.35 ± 28.69	0.002*
Thinnest	549.90 ± 25.48	563.08 ± 27.81	0.001*
Difference	7.35 ± 6.20	6.27 ± 4.33	0.124
ISV	20.38 ± 5.52	24.01 ± 6.31	0.117
IHD	0.008 ± 0.004	0.007 ± 0.006	0.817
Angle Kappa	0.23 ± 0.14	0.26 ± 0.15	0.794
Lenticule thickness	101.90 ± 12.11	136.68 ± 25.66	< 0.001*
Optic zone	6.64 ± 0.21	6.41 ± 0.22	< 0.001*
Incision length	3.73 ± 0.27	3.76 ± 0.28	0.534

BCVA: best-corrected visual acuity, CCT: central corneal thickness, IHD: index of height decentration, IOP: intraocular pressure, ISV: index of surface variance, N: number, SD: standard deviation, SE: spherical equivalent

* Denotes a significant difference between groups

**Fig. 1:**
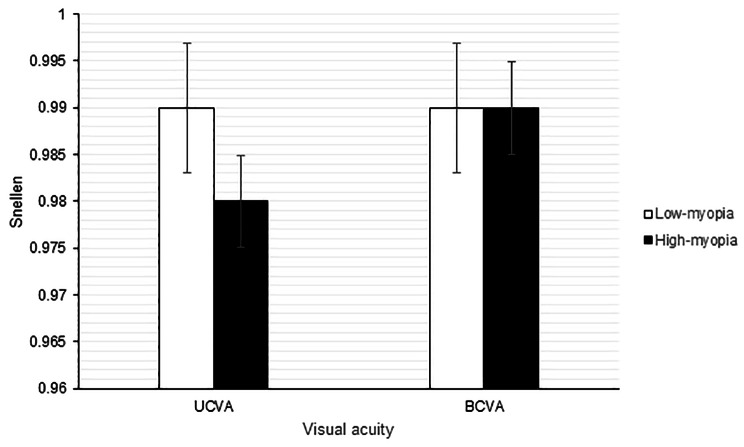
Comparison of postoperative visual acuity between the two groups

**Fig. 2:**
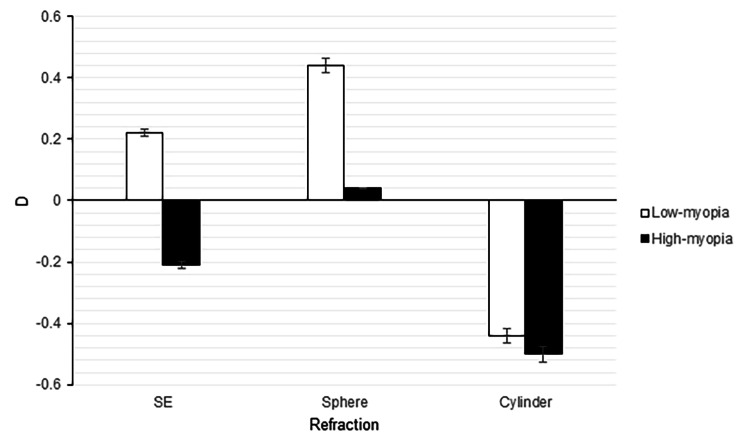
Postoperative refraction in the two groups * Denotes a significant difference between the groups

One year after SMILE surgery, the UCVA (*P* = 0.206) and BCVA (*P* = 0.963) did not significantly differ between the low myopia and high myopia groups (Fig. 1). On the other hand, the SE and sphere power were significantly more positive in the low myopia group than in the high myopia group (both *P* < 0.001), while the cylinder power was similar between the low myopia and high myopia groups (*P* = 0.298) (Fig. 2). The mean cylinder power one month after SMILE surgery was − 0.47 D, which was similar to the mean cylinder power one year after SMILE surgery (-0.48 D, *P* = 0.776). Five (6.25%) and 9 (6.00%) patients in the low myopia and high myopia groups, respectively, presented with high postoperative residual astigmatism; these differences were not significant (*P* = 0.569). There were 218, 7, and 5 participants who had within-the-rule, against-the-rule and oblique cylinder axes preoperatively, respectively, and 209, 13 and 8 participants who had within-the-rule, against-the-rule and oblique cylinder axes postoperatively, respectively. The distributions of the cylinder axis before and after SMILE surgery were similar (*P* = 0.652).

Among the potential risk factors for high postoperative residual astigmatism, the steep corneal curvature (coefficient = 0.662, 95% CI = 0.415–1.293, *P* = 0.009) and cylinder power derived from the topographic measurement (coefficient: 0.056, 95% CI: 0.002–0.487, *P* = 0.048) significantly and positively correlated to higher amount of postoperative astigmatism, while the cylinder power derived from cycloplegic refraction and other covariates did not significantly associate with higher amount of postoperative astigmatism (all *P* > 0.05). In the low myopia group, steep corneal curvature was correlated with a greater risk of high postoperative residual astigmatism (coefficient = 0.324, 95% CI = 0.112–0.735, *P* = 0.012) (Table 2). In addition, higher cycloplegic cylinder power (coefficient: 0.631, 95% CI: 0.264–3.003, *P* = 0.025), steep corneal curvature (coefficient: 1.034, 95% CI: 0.507–2.394, *P* = 0.001), greater topographic cylinder power (coefficient: 0.105, 95% CI: 0.016–1.015, *P* = 0.035), small optic zone (coefficient: -0.164, 95% CI: -0.309 - -0.081, *P* = 0.046) and longer incision length (coefficient: 0.026, 95% CI: 0.008–0.135, *P* = 0.041) were associated with a high rate of postoperative residual astigmatism in the high myopia group (Table 3).

According to the interaction test, the effects of cycloplegic cylinder power (coefficient: 0.604, 95% CI: 0.227–1.397, *P* = 0.002), topographic cylinder power (coefficient: 0.087, 95% CI: 0.034–0.167, *P* = 0.012), and longer incision length (coefficient: 0.556, 95% CI: 0.134–2.197, *P* = 0.004) on the incidence of high postoperative residual astigmatism development were greater in the high myopia group than in the low myopia group (Table 4).

**Table 2:** Risk factors for high postoperative residual astigmatism in patients with low myopia

Covariate	Coefficient	95% CI		***P***
		Lower limit	Upper limit	
Age	-0.002	-0.014	0.007	0.680
Sex	0.001	-0.153	0.222	0.733
Cycloplegic refraction				
SE	0.004	-0.070	0.104	0.863
Sphere	0.011	-0.182	0.201	0.932
Cylinder	-0.042	-0.319	0.204	0.745
Axis	-0.014	-0.208	0.179	0.874
IOP	-0.005	-0.024	0.020	0.750
BCVA	-1.379	-3.114	0.276	0.124
Manifest refraction				
SE	0.013	-0.092	0.261	0.777
Sphere	0.014	-0.172	0.207	0.882
Cylinder	0.011	-0.217	0.239	0.943
Axis	-0.006	-0.158	0.155	0.978
Steep corneal curvature	0.320	0.106	0.727	0.015*
Flat corneal curvature	0.003	-0.038	0.041	0.903
Topographic cylinder	0.032	-0.003	0.140	0.071
Topographic axis	0.092	-0.491	0.695	0.782
CCT				
Apex	0.222	-0.019	1.134	0.080
Thinnest	0.005	-0.004	0.009	0.857
Difference	-0.001	-0.012	0.011	0.923
ISV	0.097	-0.049	0.143	0.192
IHD	0.076	-0.032	0.135	0.088
Angle Kappa	0.072	-0.043	0.196	0.094
Lenticule thickness	0.005	-0.004	0.012	0.329
Optic zone	-0.038	-0.515	0.087	0.083
Incision length	-0.127	-0.382	0.124	0.333

BCVA: best-corrected visual acuity, CCT: central corneal thickness, CI: confidence interval, IHD: index of height decentration, IOP: intraocular pressure, ISV: index of surface variance, N: number, SE: spherical equivalent

* denotes a significant effect of the covariate

**Table 3:** Risk factors for high postoperative residual astigmatism in the high-myopia population

Covariate	Coefficient	95% CI		***P***
		Lower limit	Upper limit	
Age	0.000	-0.006	0.007	0.971
Sex	0.014	-0.022	0.085	0.579
Cycloplegic refraction				
SE	-0.201	-2.176	1.573	0.195
Sphere	-0.095	-0.217	0.028	0.113
Cylinder	0.632	0.260	3.027	0.024*
Axis	-0.058	-0.142	0.030	0.263
IOP	0.002	-0.014	0.016	0.878
BCVA	-0.812	-1.887	0.262	0.131
Manifest refraction				
SE	0.012	-0.050	1.103	0.247
Sphere	0.087	-0.038	0.222	0.178
Cylinder	0.055	-0.027	0.142	0.126
Axis	0.013	-0.056	0.089	0.747
Steep corneal curvature	1.032	0.500	2.311	0.003*
Flat corneal curvature	-0.001	-0.029	0.028	0.985
Topographic cylinder	0.104	0.016	1.013	0.034*
Topographic axis	-0.022	-0.122	0.078	0.733
CCT				
Apex	0.003	-0.022	0.045	0.619
Thinnest	-0.001	-0.004	0.002	0.427
Difference	-0.001	-0.011	0.008	0.790
ISV	0.106	-0.021	0.168	0.071
IHD	0.089	-0.030	0.159	0.080
Angle Kappa	0.092	-0.018	0.255	0.056
Lenticule thickness	0.004	-0.003	0.010	0.977
Optic zone	-0.162	-0.328	-0.080	0.045*
Incision length	0.024	0.006	0.133	0.044*

BCVA: best-corrected visual acuity, CCT: central corneal thickness, CI: confidence interval, IHD: index of height decentration, IOP: intraocular pressure, ISV: index of surface variance, N: number, SE: spherical equivalent

* denotes a significant effect of the covariate

**Table 4:** Specific risk factors for high postoperative residual astigmatism in high myopia population in comparison to low myopia population

Covariate (reference: low myopia group)	Coefficient	95% CI		***P***
		Lower limit	Upper limit	
Age	0.003	-0.019	0.062	0.908
Sex	-0.006	-0.099	0.076	0.820
Cycloplegic refraction				
SE	-0.001	-0.104	0.116	0.882
Sphere	0.101	-1.147	1.782	0.657
Cylinder	0.608	0.229	1.411	0.002*
Axis	-0.011	-0.109	0.038	0.431
IOP	0.016	-0.051	0.136	0.082
BCVA	0.008	-1.550	2.055	0.400
Manifest refraction				
SE	0.015	-0.026	0.073	0.902
Sphere	0.044	-0.092	0.137	0.663
Cylinder	0.085	-0.016	0.150	0.078
Axis	0.136	-0.017	0.283	0.091
Steep corneal curvature	0.078	-0.034	0.159	0.202
Flat corneal curvature	-0.008	-0.011	0.019	0.824
Topographic cylinder	0.082	0.036	0.157	0.013*
Topographic axis	-0.004	-0.147	0.233	0.661
CCT				
Apex	-0.016	-0.212	0.219	0.836
Thinnest	-0.022	-0.321	0.214	0.567
Difference	0.000	-0.013	0.013	0.999
ISV	0.009	-0.011	0.025	0.375
IHD	0.018	-0.007	0.030	0.096
Angle Kappa	0.011	-0.004	0.027	0.082
Lenticule thickness	0.001	-0.009	0.011	0.969
Optic zone	-0.094	-0.147	0.035	0.081
Incision length	0.558	0.136	2.322	0.003*

BCVA: best-corrected visual acuity, CCT: central corneal thickness, CI: confidence interval, IHD: index of height decentration, IOP: intraocular pressure, ISV: index of surface variance, N: number, SE: spherical equivalent

* Denotes a significant difference between groups

## Discussion

In the present study, the incidence of high postoperative residual astigmatism did not differ significantly between patients with low myopia and those with high myopia. In addition, a steep corneal curvature was associated with a greater risk of high postoperative residual astigmatism in patients with low myopia, and high cylinder power derived from cycloplegic refraction, high cylinder power derived from topographic measurement, steep corneal curvature, small optic zone and longer incision length were related to high postoperative residual astigmatism only in the high myopia population. In addition, high cylinder power derived from cycloplegic refraction, high cylinder power derived from topographic measurement and longer incision length are associated with a higher risk of high postoperative residual astigmatism in high myopia individuals than in low myopia individuals. Other parameters, including flat corneal curvature, IHD, ISV, and angle kappa, were not significantly associated with high postoperative residual astigmatism.

Some hypotheses about early refractive errors, including myopia regression and high postoperative residual astigmatism after corneal refractive surgeries, have been proposed [18]. Postoperative corneal epithelial thickening could be a mechanism of early myopic regression after LASIK surgery [19, 20]. In addition, postoperative dry eye disease caused by LASIK could damage the ocular surface and change the corneal epithelial structure [21]. In addition to epithelial proliferation, the forward shift in corneal curvature could be another etiology of early myopic regression [19]. The corneal strength after refractive surgeries, such as photorefractive keratectomy, LASIK and SMILE, decreases, and patients who undergo SMILE have the highest degree of postoperative corneal stiffness [22]. An impaired corneal structure could cause anterior movement of the corneal curvature, an increase in total corneal refractive power and subsequent myopic regression [23–25]. On the other hand, postoperative astigmatism after refractive surgery has some similar etiologies as early myopic regression [24, 26, 27]. In addition, posterior corneal irregularity after refractive surgery could be associated with the occurrence of postoperative astigmatism and corneal curvature changes [28]. Moreover, the location and length of the corneal incision during cataract surgery influence the degree of postoperative astigmatism [29, 30]. Since SMILE surgery is also a corneal surgery that involves a change in corneal curvature and the creation of corneal incision [8], related parameters may influence the degree of postoperative astigmatism after SMILE surgery, similar to LASIK and cataract surgery. The above concept was partially supported by the results of the present study.

The degree of steep corneal curvature was correlated with a high risk of postoperative residual astigmatism in both low myopia and high myopia populations, while the other topographic and surgical parameters were also related to a higher incidence of high postoperative residual astigmatism in the high myopia population. In a previous study, a smaller optic zone and higher degree of preoperative astigmatism were associated with the development of postoperative astigmatism after LASIK surgery [16], and a change in corneal curvature was also associated with the occurrence of postoperative astigmatism in patients who underwent photorefractive keratectomy [31]. However, few studies have evaluated the possible risk factors for high postoperative residual astigmatism after SMILE surgery [17]. To our knowledge, the present study may provide preliminary conclusions about the possible risk factors for high postoperative residual astigmatism in SMILE patients with different degrees of myopia. Although high myopia is theoretically related to a greater chance of high postoperative residual astigmatism, a more detailed analysis of this issue could be conducted. In addition, all the SMILE surgeries were performed by an experienced refractive surgeon, and the follow-up period in the present study was 1 year, which was adequate. Consequently, the integrity of the results may be acceptable. Alterations in corneal curvature are important factors that contribute to the development of astigmatism after several different types of ophthalmic surgeries [28, 32, 33], and the strong correlation between steep corneal curvature and the development of high postoperative residual astigmatism in the two groups of patients in the present study corresponded to previous findings. In the high myopia group, cycloplegic and topographic cylinder powers were associated with the development of high postoperative residual astigmatism. This may be because fine attachment of the interface takes longer in patients with high myopia with deeper stromal pockets [34]; thus, preoperative corneal irregularities may persist more easily. In addition, the small optic zone contributes to lower refractive stability [16], and a longer corneal incision could prominently alter corneal structure ^30^. These factors may cause corneal irregularity in the high myopia group. The low myopia group exhibited prominent overcorrection of spherical power, possibly due to the use of a nomogram for correcting for spherical power. A modification of our nomogram for low myopia individuals may be needed.

Cycloplegic and topographic cylinder and incision length had greater significant effects on the development of high postoperative residual astigmatism in the high myopia group than the low myopia group. This may be a relatively new finding about the risk factors for post refractory surgery astigmatism. Patients with high myopia who underwent SMILE surgery were more prone to having a longer recovery period for both visual acuity and refraction status than patients with low myopia, which was probably due to the later attachment of the interface [34]. In addition, both myopic regression and postoperative corneal curvature alterations were positively correlated with the degree of preoperative myopia in a previous study [10]. Since both high myopia and high astigmatism indicate a steeper corneal curvature, the coexistence of high preoperative myopia and astigmatism may be more markedly correlated with greater corneal irregularity and high postoperative residual astigmatism after SMILE surgery than low preoperative myopia and astigmatism. In addition, the manifest cylinder included ocular residual astigmatism, which is not correlated with corneal regularity [35, 36]; thus, the association between preoperative manifest cylinder power and high postoperative residual astigmatism may not be as strong as that between the topographic cylinder and high postoperative residual astigmatism. The length of the corneal incision is related to the degree of surgically induced astigmatism in cataract surgery patients [29, 30], and we speculate that a longer corneal incision leads to corneal instability in the early postoperative period, mainly in the high myopia population; thus, incision-related astigmatism was more prevalent in that group. However, further research is needed to prove this hypothesis.

For the efficiency and predictability of SMILE surgery in the present study, the mean UCVA of the study population one year after surgery was 0.99, and 95% of patients exhibited a UCVA > 20/25 or better. In previous research, all patients who underwent SMILE surgery exhibited a UCVA > 20/25 at two years post-surgery [37], and another article described that 82 to 96% of individuals exhibited a UCVA greater than 20/25 at three months post-surgery [38]. Thus, the satisfactory results of SMILE surgery in the present study are comparable with the findings of previous publications [37, 38]. For predictability, the SE one year post surgery was − 0.02 D in the present study, which was not inferior to the − 0.28 D to -0.38 D range of SE in previous publications [38, 39]. In addition, the mean astigmatism was − 0.48 D in the present study, which is similar to the postoperative astigmatism observed in refractive surgery patients in previous studies [40, 41]. Regarding safety, no severe complications, such as keratitis, corneal ectasia or loss of BCVA, were found in the study group. As a consequence, the surgical outcome of SMILE in the present study may be adequate.

There are still some limitations in the present study. First, the retrospective design of the present study decreased the homogeneity of the study population, which may cause bias. Second, we collected the data at only three time points since some participants did not return to our clinic during the follow-up period, and the change in postoperative astigmatism could not be assessed in detail. Moreover, several corneal biomechanical factors, including corneal hysteresis, corneal resistance factor and deformation amplitude, were not measured in the present study because the instrument was not available and because the relationship between corneal biomechanics and high postoperative residual astigmatism could not be evaluated. High myopia is always associated with a greater chance of regression, but we cannot analyze this covariate due to the high collinearity between high myopia and high myopia-related regression. Finally, the sample size in the present study was small (230 eyes), which may have contributed to the statistical bias.

## Conclusions

In conclusion, a steep corneal curvature was a universal risk factor for high postoperative residual astigmatism development in patients who underwent SMILE surgery with any degree of sphere strength. Furthermore, cycloplegic and topographic cylinder powers and corneal incision length were more strongly correlated with postoperative residual astigmatism development in the high myopia population than in the low myopia population. Consequently, patients with the above factors could be informed about the possibility of high postoperative residual astigmatism, and the surgical method for these patients could be modified. Additional large-scale prospective studies evaluating the association between corneal biomechanics and high postoperative residual astigmatism after SMILE surgery are needed.

## Data Availability

The data that support the findings of this study are available from the corresponding author upon reasonable request.

## Declarations

### Authors’ information

C.-Y.L., J.-H.S., C.-C.C. and C.-K.C. are ophthalmologists from a refractive center, J.-Y.H. is a statistical specialist, and I.-B.L. and S.-F.Y. are professors from universities.

## Abbreviations

LASIK: laser in situ keratomileusis
SMILE: small incision lenticule extraction
D: diopter
UCVA: uncorrected visual acuity
BCVA: best corrected visual acuity
IOP: intraocular pressure
CCT: central corneal thickness
SE: spherical equivalent
IHD: index of height decentration
ISV: index of surface variance
CI: 95% confidence interval
N: number
SD: standard deviation
